# Evaluating the Co‐Design and Implementation of a Multicomponent Intervention to Improve Communication in Aged Care: A Nested Process Evaluation Protocol

**DOI:** 10.1111/hex.70782

**Published:** 2026-07-25

**Authors:** Alicia J. King, Sarah J. Wallace, Lauren Fothergill, Sally Zingelman, Kyla Hudson, Peter Worthy, Michelle King, Danijela Hliš, Deirdre Fetherstonhaugh, Geoff Argus, Kirstine Shrubsole, Amy Coe, Victoria J. Palmer

**Affiliations:** ^1^ The ALIVE National Centre for Mental Health Research Translation The University of Melbourne Melbourne Australia; ^2^ The Department of General Practice and Primary Care, Melbourne Medical School The University of Melbourne Melbourne Australia; ^3^ Queensland Aphasia Research Centre, School of Health and Rehabilitation Sciences The University of Queensland Brisbane Australia; ^4^ Surgical, Treatment and Rehabilitation Service Education and Research Alliance The University of Queensland and Metro North Hospital and Health Service Brisbane Australia; ^5^ The School of Electrical Engineering and Computer Science The University of Queensland Brisbane Australia; ^6^ Conversations About Care Lived Experience Advisory Group Brisbane Australia; ^7^ Australian Centre for Evidence Based Aged Care La Trobe University Melbourne Australia; ^8^ Southern Queensland Rural Health The University of Queensland Toowoomba Australia

**Keywords:** aged care, cluster randomised controlled trial, communication, consumer engagement, health care, health services research, implementation, nursing, nursing homes, process evaluation

## Abstract

**Background:**

Experience‐based co‐design (EBCD) seeks to bring together people with lived experience of healthcare with other interest holders to collaboratively generate creative solutions.

**Objective:**

A cluster randomised controlled trial is planned to assess the feasibility, acceptability and effectiveness of a co‐designed multicomponent intervention to improve communication in aged care services.

**Design:**

This paper describes a nested process evaluation design for the EBCD and implementation of the intervention with the aim of understanding the role of co‐design and other factors in achieving impact.

**Method:**

Primary data sources will include surveys, interviews, focus groups, field notes and observations of co‐design activities. Secondary sources will include data collected and materials produced by the researchers developing the intervention.

**Participants:**

Aged care recipients; family members and significant others; direct care workers; managers; and other healthcare professionals will be engaged as co‐designers of the intervention, and as research participants in the evaluation of its co‐design and implementation.

**Main Variables Studied:**

Evaluation findings will compare experiences of co‐design, within and across informant groups, with an explanatory theoretical model of change described in previous research employing EBCD. Additionally, the role of the co‐design and other contextual factors in the intervention's impact will be organised using the RE‐AIM (Reach, Efficacy, Adoption, Implementation and Maintenance) framework.

**Discussion:**

The involvement of people who use and deliver health services in the development of complex interventions is increasingly recommended as a means to improve impact and health outcomes. However, research evidence supporting the mechanisms of impact of co‐design and related approaches in healthcare is limited.

**Conclusion:**

Nested process evaluations of co‐design are needed to support the continued theoretical and methodological development and resourcing of co‐design in healthcare.

**Patient or Public Contribution:**

A lived experience advisory group (LEAG) of older people receiving aged care services and family or other informal supporters of those receiving aged care services has provided input to the evaluation design described in this manuscript, and a member of this group has co‐authored this article. LEAG members will continue to contribute to the conduct of the evaluation as well as the analysis and dissemination of the evaluation findings.

## 1 Introduction

Engagement of health service interest holders, including people who use and deliver health services, has emerged as best practice for the development, implementation and evaluation of complex interventions [1, 2]. Amongst approaches to engagement, co‐design is proffered as an approach to the design of interventions that reflect the preferences, needs and expertise of health services users and those most impacted by an intervention or change process [3]. Key processes of co‐design include the sharing of lived experience and growing mutual understanding between interest holders as the basis for shared decision‐making about what should change or be designed together [4]. Rather than one‐off consultations, co‐design is a relational, iterative and creative process where specific outputs may be undefined at the outset.

This protocol describes a nested process evaluation of the development and implementation of a multicomponent intervention. An adaptation of Experience‐Based Co‐Design (EBCD), a co‐design method for improving health services [3], is used in the *Unspoken, Unheard, Unmet: Improving Access to Preventative Health Care through Better Conversations about Care* project (hereafter referred to as ‘*Conversations About Care’*). The two interrelated stages of EBCD are described as an information gathering stage, the purpose of which is to understand how different points in the care journey shape experiences of care (referred to as ‘touch points’), and the co‐design stage, which brings interest holders together to design solutions [3]. More recently, EBCD has been adopted in healthcare research to support the design and implementation of interventions. This has led to interest in whether co‐design may influence the reach, efficacy, adoption, implementation and maintenance of interventions [5]. The roots of EBCD sit firmly, however, in both design and quality improvement with early applications of the method within head and neck cancer settings [6, 7], and stroke recovery [8, 9]. More recently, the approach has been adopted in dementia care [10, 11], mental health, intellectual disability and aged care settings [12, 13].

Process evaluations of co‐designed interventions and their implementation are needed to establish how factors within and beyond co‐design impact the implementation and outcomes of interventions [14, 15]. Despite the growth of co‐design in healthcare research, the description of the relationship between co‐design processes and the impacts of co‐designed interventions remains elusive [14]. Evaluations of co‐design report positive social, emotional and cognitive outcomes for participants, such as mutual support and solidarity, feeling empowered and confident and acquiring knowledge and skills [16]. However, without evaluations demonstrating the positive impact of co‐design on healthcare experiences and outcomes, it is unlikely to be embedded in routine improvement and research practice [17]. Harrison et al. [15] point to a need for greater evaluation of co‐designed interventions to identify ‘latent influences such as systemic bias, [where] healthcare cultures and mindsets may be at odds with novel strategies, especially those developed for seldom‐heard and minority populations’ (p. 48–49). This includes how experiential data collected in the information gathering stages might influence implementation or strategies prioritised within co‐design and beyond.

### 1.1 Conversations About Care

In 2018, the *Australian Royal Commission into Aged Care Quality and Safety* ‐ signalled issues around poor communication in aged care services impacting health outcomes for aged care recipients (hereafter referred to as ‘care recipients’) [18]. In Australia, community (i.e., services delivered within private homes) and residential aged care services are delivered mainly by not‐for‐profit organisations and private businesses, and a small number of government‐run services. Aged care services may include assistance with activities of daily living, respite, equipment, home modifications, healthcare and accommodation [19]. The new Australian *Aged Care Act 2024* stipulates that care recipients have the right to be informed, express opinions and be heard about the aged care services they access; communicate in their preferred language or method; have open communication with, and support from, providers when issues arise; and have complaints dealt with fairly and promptly [20].

People receiving aged care services may have communication support needs due to changes in hearing, vision, speech, language and cognition associated with healthy ageing, as well as disease processes associated with ageing, such as dementia and stroke [21, 22]. Environmental barriers to communication may coalesce with age‐related communication impairments, resulting in poorer health outcomes [23, 24, 25]. Research suggests aged care workers may face challenges meeting older persons’ communication support needs, related to time pressures and skills in assessing communication needs [26, 27, 28]. Poor communication in aged care settings has been linked to poor experiences of care [29] and loneliness [30].

In addition to the individual communication support needs of care recipients, structural changes have been identified as potential contributors to poorer quality aged care [19]. In the past two decades, the proportion of qualified nurses declined and the proportion of personal care workers, who receive less training and pay, increased from 58% to 70%, in the context of increasing sector privatisation [19]. Workforce demand is increasingly met by precariously employed migrant workers [31]. As 36% of personal care workers in aged care services and 29.8% of the general Australian population, come from culturally and linguistically diverse backgrounds [32], cultural and linguistic differences between care recipients and workers may contribute barriers to communication in resource‐limited environments [33, 34, 35].

The use of co‐design in aged care research has been recommended to develop innovative solutions to identified problems [19] and improve the acceptability and feasibility of interventions [36]. Examples of co‐design in aged and dementia care research have proliferated in recent years, particularly in Australia and the United Kingdom [37, 38, 39, 40], including specifically application in the improvement of communication in aged care [41]. Despite the application of co‐design in healthcare for older persons, evaluations that focus on the process of co‐design are limited. Khalil et al. [39] reported that a co‐designed multicomponent intervention improved the delivery and outcomes of palliative care in rural settings, but did not explore the role of co‐design in shaping these outcomes. Where co‐design is described as helpful in addressing potential barriers to the usability and acceptability of smartphone applications, effects on uptake have not been systematically evaluated [38, 40]. Similarly, Anantapong et al. [37] reported positive feedback on a co‐designed decision guide by potential users; however, the impact on care was not evaluated.

Reviews of research focusing on the involvement of older people in health and social care research suggest the approach taken by researchers may be a crucial determinant of impact. Baldwin et al. [42] found both positive and negative effects on research quality and impact, recommending future research embed evaluation within the research process. Both Baldwin et al.'s [42] and Schilling and Gerhardus's [43] earlier review found that building relationships, communication and the accessibility of participation opportunities were critical to success. Conversely, limitations in resourcing have been reported as excluding care recipients from participating in co‐design [37, 44].

The current evaluation presents an opportunity to explore adaptations needed to ensure the accessibility of EBCD, as suggested by disability co‐design literature [12, 45]. In addition to communication support needs, participants from other interest holder groups may face barriers related to English language proficiency, cultural differences and education, as well as power differentials in relationships for both care recipients and employees. Examples of adaptations to date include the use of face‐to‐face experience gathering interviews in care recipients’ private homes and residential care homes, facilitated by speech pathologists; individual meetings with co‐designers prior to workshops to ensure informed consent and plan for communication support; and use of reactions and chat functions within online co‐design workshops. The evaluation described in this paper will contribute to bridging the understanding of how the approach taken by researchers using co‐design influences outcomes.

**Figure 1:**
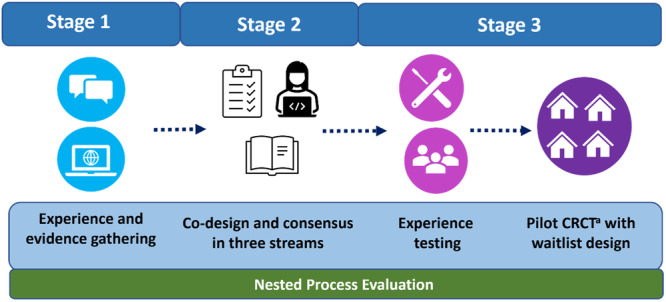
Overview of Conversations About Care stages and activities. ^a^ Cluster randomised controlled trial.

*Conversations About Care* is developing and trialling a co‐designed multicomponent intervention to improve communication in aged care settings. Figure 1 and the text that follows provide a brief overview of the project prior to describing the nested process evaluation, which is the topic of this article. Stage 2 of the project is currently underway.

#### 1.1.1 Stage 1: Experience and Evidence Gathering

In the information gathering stage (referred to in this project as ‘experience and evidence gathering’), interviews and focus groups were conducted to establish journeys experienced by people receiving and delivering community and residential aged care services, and communication‐related touch points that shaped their experiences. Interviews were conducted with care recipients with a self‐reported communication disability or difference (related to hearing, vision, speech, language, or cognitive‐communication impairments), aged 65+ years, receiving community or residential aged care services; family members and significant others; direct care workers (hereafter referred to as ‘workers’) (i.e., nurses and personal care workers); managers; and allied health care professionals working in aged care settings. A purposive sampling strategy recruited interview and focus group participants through the *Conversations About Care* partnership, research sites, investigator networks and participant referral. The recruitment approach intentionally sought diversity across culture, geography (metropolitan, regional and rural locations) and care settings (community and residential aged care facilities). Interview guides elicited experiences of communication in aged care (e.g., means and opportunities for raising concerns, positive and negative experiences of discussing care) and suggestions for best practice.

#### 1.1.2 Stage 2: Co‐Design and Consensus

Co‐design is occurring across three workstreams to develop the three intervention components: a profiling tool to identify the communication support needs and preferences (*CommSNAP*); web‐based communication partner training for workers (*CommSTAR*); and guidelines for improving communication for aged care services and regulators (*CommGuide*). The components of the intervention were selected, in preparation for co‐design, to target barriers described in the extant literature including poor understanding of care recipient communication support needs; inadequate staff skills, knowledge and confidence in culturally responsive, age‐appropriate and supportive communication; a lack of resources to support ‘communication friendly’ care planning, feedback and complaints resolution; and a lack of formalised guidance and organisational support. These barriers were mapped to the Theoretical Domains Framework [46] to identify intervention components [47], and behaviour change strategies [48] to support implementation.

A series of six co‐design workshops was convened via Zoom for the profiling tool (*CommSNAP*) and training (*CommSTAR*) workstreams. Workshops brought together all interest holder groups. They did not meet separately. Each workshop was repeated in the same week, and co‐designers had the choice of which session to attend. Early in each series, convenors presented the findings of experience gathering interviews, in the form of narrated slide shows of participant quotes illustrating touch points relating to journeys and moments that matter. Findings of scoping reviews on factors impacting communication for older adults [49] and communication partner training [50], conducted by researchers working on the project, were also presented (as relevant to each workstream) to stimulate but not limit discussion. Remaining sessions were focussed on the development and iterative review of the content, design rules and early prototypes of the intervention components’ user interfaces. Co‐design activities for the guidelines (*CommGuide*) workstream will include forums and expert panels to develop consensus based on the findings of the information gathering interviews, previous roundtable discussions, and a scoping review of existing guidelines and regulations of communication within the aged care sector in Australia, conducted by researchers on the project.

Groups represented in co‐design and experience testing activities include care recipients, family members and significant others, workers and health care professionals with expertise in communication (e.g., speech therapists, audiologists). Participants in co‐design and experience testing activities (hereafter referred to as ‘co‐designers’) are being recruited from Stage 1, and through partner organisations, including older persons advocacy groups, professional bodies and aged care services. Co‐designers will have the opportunity to participate in one or multiple workstreams and stages of the project, subject to sampling considerations.

The approach to co‐design adopted in *Conversations About Care* represents an adaptation of EBCD out of its typical focus on improvement and codesign of health services to the co‐design of a multicomponent intervention in a research context. The six stages of EBCD described by Robert et al. [51] were modified in that a greater number of experience gathering interviews (62) were conducted with aged care staff, recipients and significant others, and focus groups were also used. Experience gathering interviews were audio recorded rather than filmed, so trigger films were not shown and, due to research funding requirements, co‐designers were involved in prioritising the content of interventions rather than the choice of the three intervention components. Though the co‐design workshops were of a similar duration (3 months), the overall process has taken longer (3 years), and the celebration event is being held over until experience testing of the intervention components is complete.

#### 1.1.3 Stage 3: Experience Testing and Pilot Cluster Randomised Controlled Trial

Methods employed to gain end‐user feedback on the usability, accessibility and acceptability of the intervention components may include design walkthroughs; Think Aloud interviews [52]; joint review of prototypes by end‐users, researchers and developers against established criteria; surveys and rating scales [53, 54]; and follow‐up interviews or focus groups. The format and accessibility of the guidelines developed will be reviewed by end users with feedback incorporated.

Finally, a pilot of a cluster randomised controlled trial (cRCT) with a waitlist design will determine the feasibility and acceptability of the co‐designed multicomponent intervention. The pilot cRCT will assess the effectiveness of the intervention on care worker knowledge, self‐efficacy and attitudes; care recipient‐worker communication; and care recipient experience of care and loneliness. These outcomes have been determined by the research team, building upon previous work by the Queensland Aphasia Research Centre to understand the needs and priorities of people living with communication disabilities (i.e., improving the quality of communication between care recipients and staff). They draw upon research evidence, experiential knowledge and clinical expertise. The pilot cRCT design will be finalised following co‐design completion, and people with lived experience will be a part of that process. Participants will include workers, nurses and personal care workers, and care recipients at six residential aged care sites and four community aged care services operated by two partner organisations to the project in urban and rural locations in Australia.

### 1.2 Research Aims and Questions

Using a nested process evaluation, we are exploring how EBCD was approached to understand the design of the *Conversations About Care* intervention, the implementation factors that were identified and addressed during the trial, and the results (e.g., the outcomes of the intervention). This information will be critical to developing scaling and translation pathways for the final intervention. Detailed objectives and nested process evaluation questions are provided in Table 1.

**Table 1:** Nested process evaluation objectives and questions.

Objectives	Research questions
To describe the experiences and involvement of people within the co‐design processes for the development of the multicomponent intervention.	How did co‐designers experience the processes of intervention design, from information gathering stages through to and including experience testing and refinement?
	To what extent did the observed and reported experiences of co‐designers reflect an explanatory theoretical model of change reported in previous EBCD^a^ research?
To describe the experiences of those implementing and using the intervention and contextual factors influencing its implementation.	What contextual factors enabled and constrained the implementation of the *Conversations About Care* intervention? What worked for who, when and why?
	How did the identified contextual factors impact the reach, efficacy, adoption, implementation and maintenance^b^ of the intervention?
To describe how the co‐design of the intervention supported or led to the intended outcomes of the project around communication improvements in aged care.	What outcomes, expected and unexpected, were reported by those using the intervention following the interventions being implemented?
	How did the co‐design of the intervention impact the reach, efficacy, adoption, implementation and maintenance of the intervention?

^a^ Experience‐based co‐design.

^b^ RE‐AIM framework [5].

## 2 Methodology

This article describes a nested process evaluation for an EBCD design and implementation of the *Conversations About Care* multicomponent intervention. Process evaluations are essential to interpret results of effectiveness trials by exploring how complex interventions are implemented and received across multiple, often heterogeneous, sites [55]. Examples of process evaluations have been reported with RCTs conducted in aged care settings with [56] and without [57] the involvement of care recipients. Consistent with process evaluation methodology, we are employing a mixed‐method design, drawing upon a range of data sources and participant groups across different stages of the project. Informed by a developmental evaluation approach [58], feedback is being provided to co‐design convenors in real time during the co‐design process.

As recommended in development of complex health care interventions [59, 60], the evaluation is drawing upon two existing models described in the co‐design and implementation literature, respectively: an *Explanatory Theoretical Model of Change for Co‐Design and Co‐Production in Healthcare Improvement* [4] (hereafter referred to as ‘the explanatory theoretical model’*;* and the *RE‐AIM (reach, efficacy, adoption, implementation and maintenance) framework* [5]. The former was developed with input from lived experience co‐researchers and co‐designers in a mental health co‐design project. The lived experience advisory group and research team members in this project raised questions about its generalisability to people with communication disability (e.g., the mechanism of dialogue), which are being explored through the evaluation. The explanatory theoretical model will sensitise data analysts to the key processes and practices of EBCD, while the RE‐AIM framework will be used to organise findings regarding the impact of the co‐designed intervention.

**Figure 2:**
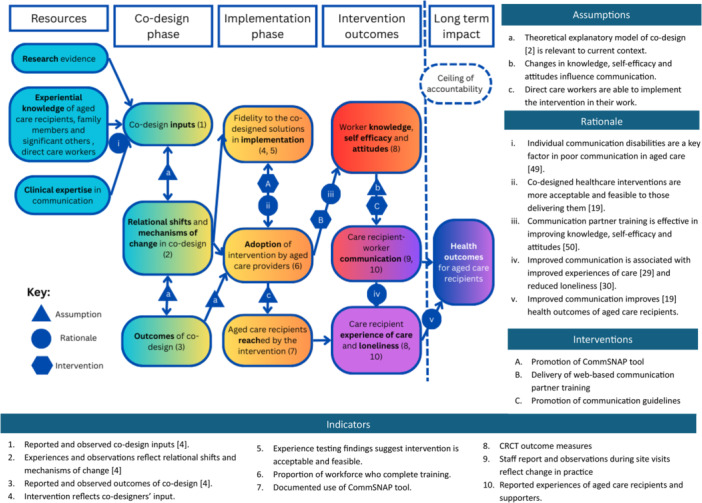
Provisional programme theory diagram representing the integration of the explanatory theoretical model of change for co‐design and co‐production in healthcare improvement.

Our approach to integrating these models within the evaluation is shown in the provisional programme theory diagram in Figure 2. The diagram also proposes the assumptions that will be explored through the evaluation, the intervention rationale, implementation‐related interventions, and process and impact indicators. Further description of these models is provided in the text that follows.

### 2.1 An Explanatory Theoretical Model of Change for Co‐Design and Co‐Production in Healthcare Improvement

**Figure 3:**
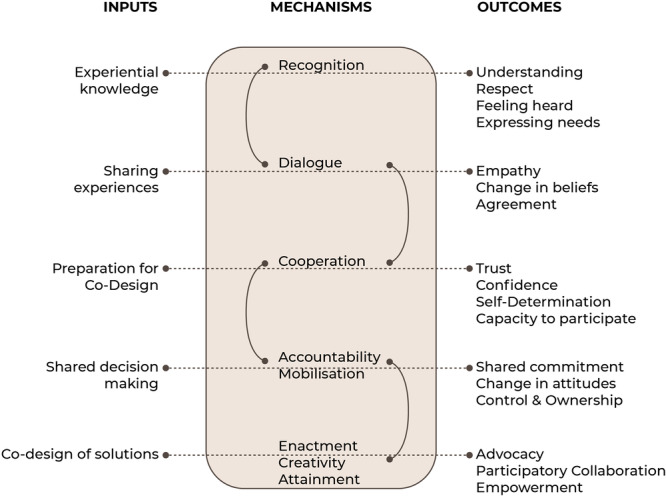
The eight mechanisms of change identified within Mental Health Experience Co‐design methodology (reproduced with the permission).

The explanatory theoretical model will provide a framework for analysis of the co‐design processes undertaken to design the *Conversations About Care* intervention and to explore the impact of co‐design on its subsequent implementation [4]. The model provides four theoretical frames to explore relational shifts (Supporting Information S1) that occur in co‐design processes, which interact with eight mechanisms of change in co‐design, shown in Figure 3. In the field of implementation science, mechanisms refer to theorised processes or events responsible for changes in outcomes when implementation strategies are employed [61]. For example, the convening team allocating a team member to monitor non‐verbal participation in co‐design discussions, such as reactions and text‐based chat (input: preparation for co‐design), may allow care recipients with communication needs to work together with other co‐designers in the co‐design process (outcome: capacity to participate) through the mechanism of cooperation. Moreover, other co‐design participants may observe how people with communication needs can be supported to engage in decision‐making processes and support the adoption of the intervention (mobilisation). Theories essential to the explanatory theoretical model, including epistemic justice, dialogical ethics and empowerment theory, will further support attention to issues of diversity and equity in the evaluation of *Conversations About Care*, such as recruitment to co‐design activities.

### 2.2 RE‐AIM Framework

Findings in relation to the impact of the co‐designed intervention beyond the co‐design group will be organised using Glasgow et al.'s [5] RE‐AIM framework, which describes the impact of an intervention as a function of five factors: reach, efficacy, adoption, implementation and maintenance. In this evaluation, the RE‐AIM framework will be used to identify process and other contextual factors influencing the observed immediate and potential longer‐term, impacts of the co‐designed *Conversations About Care* intervention.

### 2.3 Data Sources

Table 2 provides an overview of the data sources relevant to each of the project stages and the expected number of data sources. In this process evaluation, we will aim to recruit a sub‐sample of co‐designers and participants from pilot cRCT sites, across the interest holder groups, as detailed in Table 2. Given the small number of potential participants within co‐design and pilot cRCT sites and the largely qualitative methods, purposive sampling will aim for representation of a range of perspectives rather than statistical power. Further description of how this data will be collected and analysed is provided in the text that follows.

**Table 2:** Data sources for the nested process evaluation of experience‐based co‐design (EBCD) for the design and implementation of the multicomponent communication intervention by project stage.

*Conversations Aabout Care* stage	*n* a	Primary data sources	*n* a	Secondary data sources
Information gathering	62^b^	Pulse surveys of co‐designers Interviews with co‐designers Reflective field notes recorded by co‐design convenors and interviewers Structured observations of co‐design and experience testing video recordings	150^e^ 12^f^ 40^g^ 20^h^	Co‐design interview transcripts Journey maps Touchpoint presentations
Co‐design activities in workstreams	45^c^			Prototype intervention components
Experience testing				
Implementation and pilot cluster randomised controlled (cRCT) trial	150^d^	Focus groups with pilot cRCT participants and managers from pilot cRCT sites Reflective field notes recorded during site visits and after focus groups Engagement data from online intervention components	28^i^	Final intervention components Pilot cRCT outcome measures

^a^ Number of data sources, as specified.

^b^ Number of interviews and focus groups.

^c^ Care recipients, family members and significant others (*n* ≈ 15), direct care workers (*n* ≈ 15) and other interest holders (*n* ≈ 15).

^d^ Nurses (*n* ≈ 50), personal care workers (*n* ≈ 50) and care recipients (*n* ≈ 50).

^e^ Number of surveys sent.

^f^ Care recipients (*n* ≈ 3), family members and significant others (*n* ≈ 3), direct care workers (*n* ≈ 3) and other interest holders (n ≈ 3);

^g^ Number of fieldnotes.

^h^ Number of recordings.

^i^ Care recipients (*n* ≈ 6), family members and significant others (*n* ≈ 6), direct care workers (*n* ≈ 10) and managers (*n* ≈ 6).

### 2.4 Recruitment

Co‐designers are being invited to participate in the evaluation through an email link to the pulse survey, sent following each co‐design activity. Following a series of co‐design activities (i.e., workshops, roundtables, forum and expert panels) the final pulse survey will include a further invitation to participate in a research interview. Co‐designers will also have the option to be contacted by the researcher conducting the pulse survey and interviews to contact them, to complete the survey or interview over the phone.

Care recipients, significant others and staff at participating pilot cRCT sites will be recruited by research site managers. The site manager will seek consent to provide contact details (e.g., phone and/or email) to the researchers conducting the evaluation.

### 2.5 Data Collection

As summarised in Table 2, data sources informing the evaluation include primary data, collected solely for the purpose of the evaluation, and secondary data collected and produced by researchers during EBCD, experience testing and piloting of the intervention.

#### 2.5.1 Primary Data Sources

Co‐designers’ experiences are being gathered in online pulse surveys. These surveys use a five‐point Likert scale which reflects five outcomes of the mechanisms of change and a free‐text question inviting open feedback (Supporting Information S2).

Observations of all co‐design workshops and experience testing of interventions are being conducted by A.K. from video recordings of activities. A template of the inputs, mechanisms and outputs described in the explanatory theoretical model will be used to prompt but not limit reflection on the co‐design and experience testing process, with a particular focus on who is communicating, when and how (Supporting Information S3). For example, observations of co‐designers sharing experiences with the co‐design group in response to experiences shared by other co‐designers might illustrate the mechanism of dialogue, and co‐designers supporting other co‐designers to contribute by inviting their input or waiting for them to speak first might illustrate cooperation.

Co‐designer experiences are being further explored in semi‐structured interviews of maximum 60‐min duration, using an interview guide (Supporting Information S4). Interview questions cover participants’ motivations for participation; degree of involvement in project activities; positive and negative experiences of involvement; the personal impact of these experiences; and views on the intervention. Interviews are being conducted via Zoom videoconferencing or telephone, audio recorded, transcribed by a professional transcription service and checked by the researcher who conducted the interview, and an interview summary shared back to the participant.

The views of care recipients, family members and significant others, and staff at pilot cRCT sites who received the *Conversations About Care* intervention will be explored in focus groups, of no longer than 90 min. Focus groups will be streamed by informant group (i.e., care recipients, and/or family members and significant others; workers; managers) but may include participants from multiple sites. Prompt questions for all groups will include their involvement in the co‐design process (if any), impacts of the intervention, and suggestions for improvements or other changes to improve communication (Supporting Information S5–S7). Care recipients, family members and significant others will also be asked about their experiences of communication in their aged care service prior to the intervention (Supporting Information S5). Workers and managers will be asked about their use of the resources developed (Supporting Information S6 and S7). Where preferred by the participant, individual interviews will be conducted using the same questions. Focus groups (or interviews) will be conducted and recorded similarly to interviews in earlier stages 2, with the addition of an in‐person format, during visits to pilot cRCT sites.

A demographic questionnaire is being administered online or by the researcher prior to research interviews and focus groups to enable an aggregated description of evaluation participants and comparison of experiences across informant groups (Supporting Information S8). An abbreviated version of these questions is used at the start of the pulse survey (Supporting Information S6).

Reflective field notes are being recorded by the researchers facilitating co‐design activities (Supporting Information S9), and evaluation‐related interviews and focus groups (Supporting Information S10), using prompts adapted from Phillippi and Lauderdale [62] to record relevant contextual information, observations of co‐designer or participant engagement, significant discussion topics, reflections on facilitation and changes for future activities. Field notes will also be recorded during visits to pilot cRCT sites using a template adapted from Phillippi and Lauderdale [62] and the RE‐AIM framework [5], such as comments and observations relevant to the reach, effectiveness, adoption, implementation and maintenance of the intervention components (Supporting Information S11).

Engagement data from online intervention components (i.e., *CommSNAP* tool, communication partner training, guideline website) will also be used to assess quantitatively the adoption of interventions. This may include the number of times they were accessed, completion rates, time spent and navigation paths to identify where users looped or regressed within the interaction.

#### 2.5.2 Secondary Data Sources

Data collected and produced by researchers during development of the intervention, alongside the final intervention components and pilot cRCT outcome measures, will be reviewed as a means of triangulating participants’ reported experiences and perspectives, researchers’ observations and reflections, with artefacts of the co‐design process. A representative sample of each of the data sources detailed in Table 2 will be reviewed with reference to the research aims.

### 2.6 Data Analysis

#### 2.6.1 Quantitative Analysis

Due to the small sample size (see Table 2), quantitative data collected in the co‐designer survey, participant demographics and engagement with online interventions will be analysed and presented using descriptive statistics only. Comparisons of participants’ ratings of items over time, in a series of workshops, and between informant groups will be conducted to observe and consider differences relevant to the evaluation aims and questions. Demographic data will be used to describe participants and to support comparisons of quantitative and qualitative data across informant groups.

#### 2.6.2 Qualitative Data Analysis

Qualitative data analysis will adopt a framework analysis approach [63], while retaining an abductive stance whereby existing theories will inform but not limit analysis [64]. The reported experiences and observations of participants and co‐design convenors will be analysed with reference to the explanatory model while remaining open to new insights. The domains of the RE‐AIM framework [5] will also be used to consider participants’ reported experiences in terms of the impact of the intervention.

Descriptive, process and emotion coding methods described by Saldana [65] will be used to code interview and focus group transcripts, researcher field notes and observations of recordings. Descriptive, comparative and relational analysis strategies described by Miles et al. [66] will be used to describe, compare and look for relationships in the data with reference to theoretical models.

Analysis conducted by the primary researcher conducting interviews and focus groups (A.K.) will be subjected to the following review methods. Individual interview summaries will be prepared and sent to participants who agree to receive them via email or hard copy. A sub‐sample of transcripts, interview summaries and a tabular heat map of coded interviews (one from each group interviewed) will be reviewed by another team member. The second reviewer (V.P.) will also explore the data with the explanatory theoretical model and RE‐AIM frameworks, which will be discussed to explore convergence and differences in coding. To reduce potential bias in analysis, both AK and VP are employed by another institution and not involved in the facilitation of co‐design workshops or experience testing. Preliminary findings, including pattern codes, categories, themes and concept maps [67] generated throughout the data analysis process will also be reviewed, challenged and refined by the evaluation working group (see *Researcher Positionality*), fostering a collaborative approach to interpretation and researcher reflexivity by providing alternate perspectives on the data.

### 2.7 Researcher Positionality

The researchers leading the evaluation are trained in participatory design, applied ethics, and narrative (V.P.) and occupational therapy, community development and qualitative research (A.K.). Supporting the evaluation are a working group comprised of the team leading *Conversations About Care* who are trained in speech pathology (S.W., K.H., S.Z., K.S.), public health and gerontology (L.F.), law and sociology (M.K.), and interaction design (P.W.); investigators external to these teams with expertise in aged (D.F.), rural (G.A.) healthcare and implementation science (K.S.); and two lived experience advisory group (LEAG) members, between them representing care recipient, significant other, advocate, direct care worker and bicultural perspectives (D.H.).

### 2.8 Patient and Public Involvement

A LEAG has been established to provide expert feedback on key elements of *Conversations About Care*, including the nested process evaluation. LEAG members shaped the overall evaluation design and questions at an initial partners’ day workshop. The LEAG is made up of currently eight individuals with experiences of receiving (six), supporting those receiving (two), and delivering (two) aged care services, recruited via older persons advocacy groups and partnering aged care services. The LEAG is convened regularly to provide updates on the overall project and seek members’ input on questions arising during its conduct, and selected LEAG members also provide input via other project working groups. LEAG members will also provide guidance on the dissemination of the project outcomes, including evaluation findings.

In addition to their contributions to the overall project design and evaluation working group previously described, members of the LEAG have ongoing input into the design of the evaluation described in this protocol. For example, LEAG members suggested that the researcher conducting the data collection for the evaluation (A.K.) attend the first session of the co‐design workshops to introduce herself before they received the pulse survey and interview invites from her email. LEAG members also reviewed participant‐facing materials (e.g., plain language statement and accessible video, survey and interview questions). As a result, the wording of statements within the pulse survey was changed to create an accessible questionnaire, the number of questions was reduced, and an open response was added. LEAG members pilot tested the pulse survey, online consent form and demographic questionnaire instruments for usability. This resulted in, amongst other changes, the addition of text‐to‐speech buttons. One LEAG member is a co‐author of this publication. LEAG involvement will continue throughout the data collection and analysis stages of the evaluation, and findings will be reported in accordance with the Guidance for Reporting Involvement of Patients and the Public (GRIPP‐2) long‐form checklist [68].

### 2.9 Ethics and Dissemination

Ethical approval for the evaluation described in this paper has been obtained from the University of Melbourne Human Research Ethics Committee (project no. 31626). Ethical approval for *Conversations About Care* has been obtained from the University of Queensland Human Research Ethics Committee for the information gathering (2023/HE000054); co‐design workshops (2024/HE000927; 2025/HE000927); consensus‐building roundtable discussions (2024/HE001694); and experience testing (2025/HE002268; 2026/HE010597). Ethical approval for further guideline development activities and the pilot cRCT will be sought prior to commencement.

Evaluation findings will be shared at each stage of *Conversations About Care* (see Figure 1) with partner organisations via steering group meetings; with evaluation participants. An accessible overview via email or hard copy and the project website (https://shrs.uq.edu.au/research/research-centres-and-units/qarc/conversations-about-care) for participants, aged care professionals and researchers. Conference presentations, research seminars and peer‐reviewed journal articles are planned.

## 3 Discussion

This article describes a nested process evaluation protocol to address the current evidence gap for the impact of research co‐design of interventions on the reach, efficacy, adoption, implementation and maintenance of complex healthcare interventions. In this project, a multicomponent intervention to improve communication in aged care settings is the focus. While the involvement of health service users and other interest holders is increasingly mandated in research reporting and funding guidelines [1, 68], evidence for the impact of this involvement on design and implementation is limited and inconclusive [13, 69]. Building on Modigh's earlier work, Fredriksson et al.'s [70] recent conceptual review of patient and public involvement literature synthesised proposed impacts within the sub‐category of health service provision, namely: service improvement; staff knowledge and attitudes; staff satisfaction; efficient use of resources; system legitimacy; and reduced complaints. Amongst the outcomes being measured within the *Conversations About Care* pilot cRCT are aged care worker knowledge, self‐efficacy and attitudes, as well as outcomes related to measures of service improvement named by Fredriksson, such as quality of care and responsiveness to needs and complaints (i.e., care recipient‐worker communication and care recipient experience of care and loneliness). As well as encouraging healthcare researchers to consider their choice of outcomes and how to measure them, Fredriksson et al. [70] identified ‘process measures’ as a priority for future research, due to a lack of theoretical exploration concerning the outcomes or impacts of patient and public involvement within the ‘predominantly empirical health science literature’ (p. 9). Process evaluations are commonly accepted as a means of assessing the fidelity and quality of implementation, mechanisms of action within interventions, and context in healthcare intervention research [71], and are consistent with a theory‐based approach supported in recent guidance for health services research [54, 72]. Grant et al. [73] cautioned researchers to be selective but explicit in their choice of evaluation aims and methods to pursue them. Of the potential domains they described for evaluation of cRCTs, our evaluation will explore the recruitment and response of clusters; the recruitment, reach and response of individuals; the theory used to the develop the intervention; and the context in which the trial is being conducted, using a combination of participant reported, observational and online engagement data within the context of this being a pilot stage of work for the cRCT.

The use of qualitative or mixed methods designs has been recommended in the development and evaluation of health interventions, ‘…going beyond asking whether an intervention works’ [1] to explore, describe, explain and predict how interventions work in the real world [1, 74]. Focus groups and interviews with end users and site visits following implementation of the *Conversations About Care* intervention will allow us to go beyond the effectiveness of the intervention for those who used it to understand other factors influencing its impact.

A key challenge in the application of process evaluations, is that they often occur after intervention design, overlooking the design process and how it influences outcomes and implementation [13]. The role of co‐design and related approaches, particularly how they are approached, in achieving positive impacts on health services is frequently neglected in evaluations of healthcare interventions [13, 71]. Using the metaphor of a tree, Peters et al. [14] suggested evaluation of a research co‐design process might start ‘below ground’ with the context within the co‐design group (the roots of the tree) and progress ‘above ground’ to the wider context outside of the co‐design group (the branches and leaves of the tree) (p. 10). ‘Below ground’ within this evaluation will be primarily explored through the lens of an explanatory theoretical model, while ‘above ground’ outcomes will be related to the domains of the RE‐AIM framework; the two are interconnected since foundations that are premised on lived experience driving the intervention design ought to shape what emerges above ground. Use of a nested process evaluation design throughout all stages of the project will allow us to understand how ‘below ground’ processes within the EBCD bore (or failed to bear) fruit in the implementation and outcomes of the intervention.

As highlighted by Peters et al. [14], the mechanisms of change of co‐design are poorly described in most evaluations. Beyond understanding the findings of the pilot cRCT, the nested process evaluation will build our theoretical understanding of the mechanisms of change operating within co‐design. It will do this by application of an explanatory theoretical model for co‐design adapted within the aged care context. Concerns regarding the accessibility of co‐design [11, 44, 45] to people with age‐related disabilities have been raised by multiple authors. The evaluation provides the opportunity to describe how factors such as the accessibility of co‐design processes impact the uptake and outcomes of interventions.

In addition to exploring the process of co‐design, the evaluation further presents an opportunity to explore contextual factors impacting the use of co‐design within the aged care setting in Australia, which is a complex environment with multiple interest holder groups. The importance of support from interest holders such as organisational leaders and funders is highlighted by the additional resourcing co‐design and related approaches require [16, 44, 45]. While acknowledging the value of these approaches, researchers report gaps in researcher training, time and technical assistance [14]. Examples of well‐resourced service user involvement in RCT research report benefits in building capacity and in the dissemination of findings [75], but stop short of linking co‐design processes with ‘real world’ contexts and impacts. The evaluation will relate the co‐design process and contextual factors to measures of impact described in the RE‐AIM framework [56] with the aim of providing evidence for resourcing of co‐design approaches in healthcare research. Moreover, the use of programme theory integrating process, contextual factors and outcomes will support the evaluation design and our understanding of the evaluation findings [58, 59].

Co‐design involving service users and other interest holders is promoted as the means of ensuring complex health interventions are fit‐for‐purpose, thus achieving ‘real world’ impact. However, gaps exist in our understanding of how what happens in co‐design shapes the implementation and experience of healthcare interventions to deliver on this promise, particularly since few evaluations follow the design to implementation stages of the research activities. The evaluation design described seeks to address gaps in our understanding of the value and conduct of inclusive and meaningful co‐design towards better healthcare outcomes.

## 4 Project Status

Co‐design and experience testing for different intervention components are currently underway, with the pilot cRCT commencing in early 2027. Evaluation of intervention impact will occur up to 6 months following implementation, depending on the outcome being measured. Specifically, impact on worker knowledge, attitudes and confidence will be measured after using intervention components, but the reach, adoption, implementation and impact on experience of care and loneliness will be assessed later. Assessment of intervention maintenance will be the focus of future research evaluations.

## Author Contributions

**Alicia King:** methodology (equal), writing – original draft preparation (lead), writing – review and editing (lead). **Sarah Wallace:** conceptualisation (supporting), funding acquisition (lead), methodology (supporting), project administration (lead), supervision (supporting), writing – review and editing (supporting). **Lauren Fothergill:** writing – review and editing (supporting). **Sally Zingelman:** writing – review and editing (supporting). **Kyla Hudson:** methodology (supporting), writing – review and editing (supporting). **Peter Worthy:** writing – review and editing (supporting). **Michelle King:** methodology (supporting), writing – review and editing (supporting). **Danijela Hliš:** methodology (supporting), writing – review and editing (supporting). **Deirdre Fetherstonhaugh:** methodology (supporting), writing – review and editing (supporting). **Geoff Argus:** methodology (supporting), writing – review and editing (supporting). **Kirstine Shrubsole:** methodology (supporting), writing – review and editing (supporting). **Amy Coe:** writing – original draft preparation (supporting), writing – review and editing (supporting). **Victoria Palmer:** conceptualisation (lead), funding acquisition (supporting), methodology (equal), project administration (supporting), supervision (lead), writing – original draft preparation (supporting), writing – review and editing (supporting).

## Ethics Statement

Ethical approval for the evaluation described in this paper has been obtained from the University of Melbourne Human Research Ethics Committee (approval no. 31626).

## Consent

This manuscript describes research not yet conducted. Informed verbal and/or written consent will be sought from all participants prior to participation.

## Conflicts of Interest

The authors declare no conflicts of interest.

## Permission to Reproduce Material From Other Sources

Permission has been granted by the copyright holder for reproduced material and credited to the original source.

## Supporting information


Supporting File 1

Supporting File 2

Supporting File 3

Supporting File 4

Supporting File 5

Supporting File 6

Supporting File 7

Supporting File 8

Supporting File 9

Supporting File 10

Supporting File 11

## Data Availability

Due to the sensitive and identifiable nature of the data collected in this qualitative evaluation, the original dataset will not be made publicly available.

## References

[1] K. Skivington , L. Matthews , S. A. Simpson , et al., “A New Framework for Developing and Evaluating Complex Interventions: Update of Medical Research Council Guidance,” BMJ 374 (2021): n2061, 10.1136/bmj.n2061. 34593508 PMC8482308

[2] K. Godhwani , A. K. Saka , V. Ramasamy , et al., “Deliberative Dialogue for Co‐Design, Co‐Implementation and Co‐Evaluation of Health‐Promoting Interventions: A Scoping Review Protocol,” Research Involvement & Engagement 11, no. 1 (2025): 1–7, 10.1186/s40900-025-00680-9. 40022206 PMC11869413

[3] P. Bate and G. Robert , Bringing User Experience to Healthcare Improvement: the Concepts, Methods and Practices of Experience‐based Design (Radcliffe Publishing Ltd, 2008).

[4] V. J. Palmer , W. Weavell , R. Callander , et al., “The Participatory Zeitgeist: An Explanatory Theoretical Model of Change in an Era of Coproduction and Codesign in Healthcare Improvement,” Medical Humanities 45, no. 3 (2019): 247, 10.1136/medhum-2017-011398. 29954854 PMC6818522

[5] R. E. Glasgow , T. M. Vogt , and S. M. Boles , “Evaluating the Public Health Impact of Health Promotion Interventions: The RE‐AIM Framework,” American Journal of Public Health 89, no. 9 (1999): 1322–1327, 10.2105/ajph.89.9.1322. 10474547 PMC1508772

[6] J. Hiatt , A. Young , T. Brown , M. Banks , and J. Bauer , “Improving Patient and Carer Access to Information and Support Through Head and Neck Cancer Treatment and Survivorship Using Experience‐Based Co‐Design,” Journal of Human Nutrition & Dietetics 36, no. 2 (2023): 443–452, 10.1111/jhn.13099. 36218063

[7] G. C. Brady , J. Goodrich , and J. W. G. Roe , “Using Experience‐Based Co‐Design to Improve the Pre‐Treatment Care Pathway for People Diagnosed With Head and Neck Cancer,” Supportive Care in Cancer 28, no. 2 (2020): 739–745, 10.1007/s00520-019-04877-z. 31139929

[8] M. N. Marwaa , S. Guidetti , C. Ytterberg , and H. K. Kristensen , “Using Experience‐Based Co‐Design to Develop Mobile/Tablet Applications to Support a Person‐Centred and Empowering Stroke Rehabilitation,” Research Involvement and Engagement 9, no. 1 (2023): 69, 10.1186/s40900-023-00472-z. 37620982 PMC10463694

[9] F. Jones , K. Gombert , D. Clarke , et al., “CREATE ‘Collaborative Rehabilitation Environments in Acute Stroke’ ‐ An Experience‐Based Co‐Design Approach (EBCD) to Improving Activity Experiences of Stroke Patients in 4 Hospitals in England,” International Journal of Stroke 14, no. S4 (2019): 10.

[10] A. Macdonald , K. Kuberska , N. Stockley , and B. Fitzsimons , “Using Experience‐Based Co‐Design (EBCD) to Develop High‐Level Design Principles for a Visual Identification System for People With Dementia in Acute Hospital Ward Settings,” BMJ Open 13, no. 5 (2023): e069352, 10.1136/bmjopen-2022-069352. PMC10174034 37164451

[11] S. Fiona , B. Lizzy , E. Ruth , et al., “Adapting a Dutch Web‐Based Intervention to Support Family Caregivers of People With Dementia in the UK Context: Accelerated Experience‐Based Co‐Design,” JMIR Formative Research: JMIR Publications 8 (2024): e52389, 10.2196/52389. PMC11153978 38776139

[12] M. Heerings , H. van de Bovenkamp , M. Cardol , and R. Bal , “Ask Us! Adjusting Experience‐Based Codesign to Be Responsive to People With Intellectual Disabilities, Serious Mental Illness or Older Persons Receiving Support With Independent Living,” Health Expectations 25, no. 5 (2022): 2246–2254, 10.1111/hex.13436. 35178839 PMC9615044

[13] V. J. Palmer , P. Chondros , J. Furler , et al., “The CORE Study—An Adapted Mental Health Experience Codesign Intervention to Improve Psychosocial Recovery for People With Severe Mental Illness: A Stepped Wedge Cluster Randomized‐Controlled Trial,” Health Expectations 24, no. 6 (2021): 1948–1961, 10.1111/hex.13334. 34350669 PMC8628597

[14] S. Peters , L. Guccione , J. Francis , et al., “Evaluation of Research Co‐Design in Health: A Systematic Overview of Reviews and Development of a Framework,” Implementation Science 19, no. 1 (2024): 63, 10.1186/s13012-024-01394-4. 39261956 PMC11391618

[15] R. Harrison , E. Ni She , and D. Debono , “Implementing and Evaluating Co‐Designed Change in Health,” Journal of the Royal Society of Medicine 115, no. 2 (2022): 48–51, 10.1177/01410768211070206. 35049393 PMC8902830

[16] L. Ryan , L. Hattingh , J. Carlini , et al., “Consumer Involvement in Health Service Research: A Cross‐Sectional Survey of Staff in an Australian Public Hospital and Health Service,” Australian Health Review 49, no. 1 (2025): 1–9, 10.1071/AH24186. 39183053

[17] T. Dimopoulos‐Bick , D. Follent , C. Kostovski , et al., “Finding Your Way ‐ A Shared Decision Making Resource Developed by and for Aboriginal People in Australia: Perceived Acceptability, Usability, and Feasibility,” Patient Education and Counseling 115 (2023): 107920, 10.1016/j.pec.2023.107920. 37531789

[18] Australian Government . *Royal Commission Into Aged Care Quality and Safety Final Report ‐ Volume 2: The Current System* (2018).

[19] Australian Government . *Royal Commission Into Aged Care Quality and Safety Final Report ‐ Volume 1: Summary and Recommendations* (2018).

[20] Australian Government . Aged Care Act. In: Commission ACQaS, editor. 2024.

[21] K. M. Yorkston , M. S. Bourgeois , and C. R. Baylor , “Communication and Aging,” Physical Medicine and Rehabilitation Clinics of North America 21, no. 2 (2010): 309–319, 10.1016/j.pmr.2009.12.011. 20494279 PMC3074568

[22] D. M. Guthrie , J. G. S. Davidson , N. Williams , et al., “Combined Impairments in Vision, Hearing and Cognition Are Associated With Greater Levels of Functional and Communication Difficulties Than Cognitive Impairment Alone: Analysis of InterRAI Data for Home Care and Long‐Term Care Recipients in Ontario,” PLoS One 13, no. 2 (2018): e0192971, 10.1371/journal.pone.0192971. 29447253 PMC5814012

[23] P. L. Liu , L. Zhang , X. Ma , and X. Zhao , “Communication Matters: The Role of Patient‐Centered Communication in Improving Old Adults’ Health Competence and Health Outcomes,” Health Communication 39, no. 2 (2024): 363–375, 10.1080/10410236.2023.2166209. 36628509

[24] A. Dowling , S. Garratt , and E. Manias , “Experiences and Perceptions of Medication Management Communication During Transitions of Care for Residents in Aged Care Homes and Their Caregivers: A Qualitative Meta‐Synthesis,” Journal of Clinical Nursing 34, no. 4 (2025): 1432–1451, 10.1111/jocn.17438. 39370545 PMC11933520

[25] M. Y. Savundranayagam , M. L. Hummert , and R. J. Montgomery , “Investigating the Effects of Communication Problems on Caregiver Burden,” Journals of Gerontology. Series B, Psychological Sciences and Social Sciences 60, no. 1 (2005): S48–S55, 10.1093/geronb/60.1.s48. 15643047

[26] J. M. Brooks Carthon , J. Rearden , D. Pancir , K. Gamble , and H. Rothwell , ““They're on the Fast Track”: Older Blacks Describe Experiences of Nursing Care Quality During Hospitalization,” Clinical Nursing Research 26, no. 5 (2017): 557–575, 10.1177/1054773816674478. 27836934 PMC5425319

[27] J. A. Knopp‐Sihota , L. Niehaus , J. E. Squires , P. G. Norton , and C. A. Estabrooks , “Factors Associated With Rushed and Missed Resident Care in Western Canadian Nursing Homes: A Cross‐Sectional Survey of Health Care Aides,” Journal of Clinical Nursing 24, no. 19–20 (2015): 2815–2825, 10.1111/jocn.12887. 26177787

[28] C. L. Williams , “What Spouse Caregivers Know About Communication in Alzheimer's Disease: Development of the AD Communication Knowledge Test,” Issues in Mental Health Nursing 32, no. 1 (2011): 28–34, 10.3109/01612840.2010.521292. 21208050

[29] J. Khadka , J. Ratcliffe , G. Chen , et al., A New Measure of Quality of Care Experience in Aged Care: Psychometric Assessment and Validation of the Quality of Care Experience (QCE) Questionnaire (Caring Futures Institute, Flinders University, 2020).

[30] S. A. Alsubheen , A. Oliveira , R. Habash , R. Goldstein , and D. Brooks , “Systematic Review of Psychometric Properties and Cross‐Cultural Adaptation of the University of California and Los Angeles Loneliness Scale in Adults,” Current Psychology 42 (2021): 1–15, 10.1007/s12144-021-02494-w. 34785877 PMC8586628

[31] S. Charlesworth and L. Isherwood , “Migrant Aged‐Care Workers in Australia: Do They Have Poorer‐Quality Jobs Than Their Locally Born Counterparts,” Ageing & Society 41, no. 12 (2021): 2702–2722, 10.1017/S0144686X20000525.

[32] Australian Government . *Aged Care Workforce Census Report*. 2020.

[33] S. J. Runci , B. J. Eppingstall , E. S. van der Ploeg , G. Graham , and D. W. O'Connor , “The Language Needs of Residents From Linguistically Diverse Backgrounds in Victorian Aged Care Facilities,” Australasian Journal on Ageing 34, no. 3 (2015): 195–198, 10.1111/ajag.12200. 26059466

[34] D. Gillham , A. De Bellis , L. Xiao , et al., “Using Research Evidence to Inform Staff Learning Needs in Cross‐Cultural Communication in Aged Care Homes,” Nurse Education Today 63 (2018): 18–23, 10.1016/j.nedt.2018.01.007. 29407255

[35] P. Nichols , B. Horner , and K. Fyfe , “Understanding and Improving Communication Processes in an Increasingly Multicultural Aged Care Workforce,” Journal of Aging Studies 32 (2015): 23–31, 10.1016/j.jaging.2014.12.003. 25661853

[36] *3B Royal Commission Into Aged Care Quality and Safety Final Report ‐ Volume 3B: The New System* (2018).

[37] K. Anantapong , A. Bruun , A. Walford , et al., “Co‐Design Development of a Decision Guide on Eating and Drinking for People With Severe Dementia During Acute Hospital Admissions,” Health Expectations 26, no. 2 (2023): 613–629, 10.1111/hex.13672. 36647692 PMC10010093

[38] S. Fox , L. J. E. Brown , S. Antrobus , et al., “Co‐Design of a Smartphone App for People Living With Dementia by Applying Agile, Iterative Co‐Design Principles: Development and Usability Study,” JMIR mHealth and uHealth 10, no. 1 (2022): e24483, 10.2196/24483. 35029539 PMC8800089

[39] H. Khalil , R. Hardman , M. Livens , et al., “Development and Implementation of the Palliative Care Assessment Toolkit for Rural Aged Care Facilities in Australia,” Journal of Palliative Medicine 28, no. 3 (2025): 302–309, 10.1089/jpm.2024.0368. 39745342

[40] S. Rathnayake , W. Moyle , C. Jones , and P. Calleja , “Co‐Design of an mHealth Application for Family Caregivers of People With Dementia to Address Functional Disability Care Needs,” Informatics for Health and Social Care 46, no. 1 (2021): 1–17, 10.1080/17538157.2020.1793347. 32706282

[41] M. Richards , K. Honner , J. Smith , et al. *Adapting “Listen N Talk”: Developing a Personalized Communication App with Culturally and Linguistically Diverse Residents in Aged Care*. 2025. 10.2147/CIA.S503300 PMC11889994 40060275

[42] J. N. Baldwin , S. Napier , S. Neville , and V. A. Wright‐St Clair , “Impacts of Older People's Patient and Public Involvement in Health and Social Care Research: A Systematic Review,” Age and Ageing 47, no. 6 (2018): 801–809, 10.1093/ageing/afy092. 29939208

[43] I. Schilling and A. Gerhardus , “Methods for Involving Older People in Health Research‐A Review of the Literature,” International Journal of Environmental Research and Public Health 14, no. 12 (2017): 1476, 10.3390/ijerph14121476. 29186055 PMC5750895

[44] J. van Hoof , M. H. Wetzels , A. M. C. Dooremalen , et al., “Exploring Innovative Solutions for Quality of Life and Care of Bed‐Ridden Nursing Home Residents Through Codesign Sessions,” Journal of Aging Research 2015(2015): 185054, 10.1155/2015/185054. 26543647 PMC4620296

[45] J. J. Carey , A. Spittle , C. Imms , et al., “Adapting Experience‐Based Co‐Design to Disability Research: Co‐Producing the CycLink Co‐Design Study,” Health Expectations 28, no. 3 (2025): e70276, 10.1111/hex.70276. 40296433 PMC12037989

[46] J. Cane , D. O'Connor , and S. Michie , “Validation of the Theoretical Domains Framework for Use in Behaviour Change and Implementation Research,” Implementation Science: IS 7 (2012): 37, 10.1186/1748-5908-7-37. 22530986 PMC3483008

[47] J. Isaksen and L. R. Jensen , “The (S)CAse of Denmark: Multisite Implementation of Supported Conversation for Adults With Aphasia (SCA^TM^),” Aphasiology 32, no. S1 (2018): 101, 10.1080/02687038.2018.1487015.

[48] S. Michie , M. Richardson , M. Johnston , et al., “The Behavior Change Technique Taxonomy (v1) of 93 Hierarchically Clustered Techniques: Building an International Consensus for the Reporting of Behavior Change Interventions,” Annals of Behavioral Medicine 46, no. 1 (2013): 81–95, 10.1007/s12160-013-9486-6. 23512568

[49] A. V. Manchha , B. Burton , S. Siyambalapitiya , et al., “Factors Influencing Communication for Older Adults in Residential and Community Aged Care: A Scoping Review,” Gerontologist 65, no. 7 (2025): gnaf140, 10.1093/geront/gnaf140. 40411477 PMC12314601

[50] B. Burton , K. Shrubsole , A. Manchha , M. King , and S. J. Wallace , “Communication Partner Training for Aged‐Care Workers: A Scoping Review,” International Journal of Language & Communication Disorders 60, no. 2 (2025): e70016, 10.1111/1460-6984.70016. 39977832 PMC11842017

[51] G. Robert , J. Cornwell , L. Locock , A. Purushotham , G. Sturmey , and M. Gager , “Patients and Staff as Codesigners of Healthcare Services,” BMJ: British Medical Journal 350 (2015): g7714, 10.1136/bmj.g7714. 25670179

[52] S. Mv , The Think Aloud Method: A Practical Guide to Modelling Cognitive Processes, eds. Y. F. Barnard and J. A. C. Sandberg (Academic Press, 1994).

[53] J. Brooke , “SUS: A ‘Quick and Dirty’ Usability Scale,” in Usability Evaluation in Industry, eds. P. W. Jordan , B. Thomas , I. L. McClelland , and B. Weerdmeester (CRC Press, 1996) 6, 1st ed., chap 21.

[54] V. Venkatesh , M. G. Morris , G. B. Davis , and F. D. Davis , “User Acceptance of Information Technology: Toward a Unified View,” MIS Quarterly 27, no. 3 (2003): 425–478, 10.2307/30036540.

[55] A. Oakley , V. Strange , C. Bonell , E. Allen , and J. Stephenson , “Process Evaluation in Randomised Controlled Trials of Complex Interventions,” BMJ 332, no. 7538 (2006): 413, 10.1136/bmj.332.7538.413. 16484270 PMC1370978

[56] R. Lawton , J. Murray , R. Baxter , et al., “Evaluating an Intervention to Improve the Safety and Experience of Transitions From Hospital to Home for Older People (Your Care Needs You): A Protocol for a Cluster Randomised Controlled Trial and Process Evaluation,” Trials 24, no. 1 (2023): 671, 10.1186/s13063-023-07716-z. 37838678 PMC10576890

[57] A. Söderman , M. Hälleberg Nyman , C. Werkander Harstäde , B. Johnston , and K. Blomberg , “Grasping a New Approach to Older Persons’ Dignity: A Process Evaluation of the Swedish Dignity Care Intervention in Municipal Palliative Care,” Scandinavian Journal of Caring Sciences 38, no. 2 (2024): 496–511, 10.1111/scs.13222. 37882233

[58] M. Q. Patton , Developmental Evaluation: Applying Complexity Concepts to Enhance Innovation and Use (Guilford Press, 2011).

[59] M. J. De Silva , E. Breuer , L. Lee , et al., “Theory of Change: A Theory‐Driven Approach to Enhance the Medical Research Council's Framework for Complex Interventions,” Trials 15, no. 1 (2014): 267, 10.1186/1745-6215-15-267. 24996765 PMC4227087

[60] A. O'Cathain , L. Croot , E. Duncan , et al., “Guidance on How to Develop Complex Interventions to Improve Health and Healthcare,” BMJ Open 9, no. 8 (2019): e029954, 10.1136/bmjopen-2019-029954. PMC6701588 31420394

[61] C. C. Lewis , P. Klasnja , B. J. Powell , et al., “From Classification to Causality: Advancing Understanding of Mechanisms of Change in Implementation Science,” Perspective. Frontiers in Public Health 6 (2018): 136, 10.3389/fpubh.2018.00136. 29868544 PMC5949843

[62] J. Phillippi and J. Lauderdale , “A Guide to Field Notes for Qualitative Research: Context and Conversation,” Qualitative Health Research 28, no. 3 (2018): 381–388, 10.1177/1049732317697102. 29298584

[63] M. Wensing and R. Poß‐Doering , “Process Evaluation in Health Services Research,” in Foundations of Health Services Research, eds. M. Wensing and C. Ullrich (Springer, 2023), 165–175:chap 13.

[64] S. Timmermans and I. Tavory , “Theory Construction in Qualitative Research: From Grounded Theory to Abductive Analysis,” Sociological Theory 30, no. 3 (2012): 167–186, 10.1177/0735275112457914.

[65] J. Saldana , The Coding Manual for Qualitative Researchers, 4th ed. (SAGE Publications, 2009).

[66] M. B. Miles , M. Ha , and J. Saldana , Qualitative Data Analysis: A Methods Sourcebook, 4th ed. (Sage Publications, 2018).

[67] P. Bazeley , Qualitative Data Analysis: Practical Strategies, 2nd ed. (SAGE Publications Ltd, 2021).

[68] S. Staniszewska , J. Brett , I. Simera , et al., “GRIPP2 Reporting Checklists: Tools to Improve Reporting of Patient and Public Involvement in Research,” Research Involvement and Engagement 3 (2017): 13, 10.1186/s40900-017-0062-2. 29062538 PMC5611595

[69] A. Modigh , F. Sampaio , L. Moberg , and M. Fredriksson , “The Impact of Patient and Public Involvement in Health Research Versus Healthcare: A Scoping Review of Reviews,” Health Policy 125, no. 9 (2021): 1208–1221, 10.1016/j.healthpol.2021.07.008. 34376328

[70] M. Fredriksson , F. P. Sampaio , and L. Moberg , “The Impact of Patient and Public Involvement in Healthcare Services: A Conceptual Review Spanning Social Sciences and Health Sciences,” SSM ‐ Qualitative Research in Health 7 (2025): 100517, 10.1016/j.ssmqr.2024.100517.

[71] G. F. Moore , S. Audrey , M. Barker , et al., “Process Evaluation of Complex Interventions Medical Research Council Guidance,” BMJ: British Medical Journal 350 (2015): h1258, 10.1136/bmj.h1258. 25791983 PMC4366184

[72] M. Wensing and C. Ullrich , “Use of Theories in Health Service Research,” in Foundations of Health Services Research, eds. M. Wensing and C. Ullrich (Springer, 2023), 37–47:chap 3.

[73] A. Grant , S. Treweek , T. Dreischulte , R. Foy , and B. Guthrie , “Process Evaluations for Cluster‐Randomised Trials of Complex Interventions: A Proposed Framework for Design and Reporting,” Trials 14 (2013): 15, 10.1186/1745-6215-14-15. 23311722 PMC3600672

[74] S. Hales , A. Lesher‐Trevino , N. Ford , D. Maher , A. Ramsay , and N. Tran , “Reporting Guidelines for Implementation and Operational Research,” Bulletin of the World Health Organization 94, no. 1 (2016): 58–64, 10.2471/BLT.15.167585. 26769997 PMC4709804

[75] J. Steven , R. Lisa , B. Christine , et al. Reducing Relapse and Suicide in Bipolar Disorder: Practical Clinical Approaches to Identifying Risk, Reducing Harm and Engaging Service Users in Planning and Delivery of Care—The PARADES (Psychoeducation, Anxiety, Relapse, Advance Directive Evaluation and Suicidality) Programme. Programme Grants for Applied Research: NIHR Journals Library; 2018. 30222285

