# Supplementary material for: Evaluating the Co‐Design and Implementation of a Multicomponent Intervention to Improve Communication in Aged Care: A Nested Process Evaluation Protocol
Source: Health Expect. 2026 Jul 25;29(4):e70782. doi: 10.1111/hex.70782 (PMC13401143; doi:10.1111/hex.70782)
Supplement: Supplementary file 1 — Supporting File 1 [file HEX-29-e70782-s009.docx]

| Mechanisms of change | Relational transitions | Explanatory theories |
| --- | --- | --- |
| Recognition | ‘I’—‘Them’  ► Isolated/vulnerable  ► Introspective/introverted  ► Burdened/self-focus  ► Underappreciated/not valued  ► Overlooked/invisible/fallen through the cracks/not listened to  ► Marginalised/stigmatised  ► Discriminated against  ► Harmed  ► Targeted  ► Separated | Narrative theory and epistemic justice:  Stories are central to identity formation and how individuals and others understand experiences. Storytelling enables experiential knowledge to be shared among willing listeners. It creates conditions of engagement with a problem or experience. The mechanism of recognition is an important part of storytelling—experiential knowledge must be afforded equal weight to other kinds of positivist knowledge—it must be recognised as being of value to all people involved. Experience-based co-design begins with the idea that sharing stories of experience is important to redesign and improvement efforts; it is the way that people talk about their experiences of coming in touch with different parts of a service. Here, narrative identity and epistemic justice (the question of whose knowledge is seen to count) provide the theoretical underpinning to unpack how recognition of experience and identities is facilitated or not in co-design work. |
| Dialogue | ‘Them’—‘I’—‘You’  ►Not alone/others with similar experience  ► Disbelief about experiences  ► Appreciating another’s point of view  ► Building greater understanding of institutional/environmental constraints  ► Feeling listened to/heard  ► Building a community of shared understanding | Dialogical ethics and narrative contract:  Sharing stories is dependent on dialogue between people and dialogue is also an important mechanism for developing agreements and shared understanding about experiences; a narrative contract. Dialogue is important in co-design work because it is part of the interaction with others that is needed to make decisions about improvement areas, agree on choices and build creative solutions. Through dialogue it is possible for service users and carers to recognise that some experiences are shared, staff recognise the need to value experience—sometimes dialoguing enables a difference of perspective to emerge and to allow different priorities to emerge. Service users and carers can also develop understanding of the structural constraints for staff in achieving change. Dialogue is not always facilitative it can hinder, be conflictual and people can disagree, but it is viewed as an underlying process for achieving shared understanding which makes it an essential mechanism. Here, dialogical ethics provides the theoretical underpinning to unpack this mechanism and relational work. |
| Cooperation  Accountability  Mobilisation | You’—‘Us’  ► Focus on others instead of I  ► Perceptual broadening  ► Feeling valued for experience  ► Feeling safe and confident to speak up  ► Adoption of role as change agent  ► Improvement in self-esteem | Cooperative theory:  As experience-based co-design progresses people form into groups where there is shared knowledge of multiple experiences and roles. The mechanism of cooperation comes into effect here where a sense of solidarity for a common cause evolves; without which people cannot agree on improvement areas or solutions that need to be implemented— this means that co-design can be thwarted. In the ideal, if cooperation is facilitated, people develop a sense of working together and build a shared agenda for change. Here, the mechanism of accountability enables the collective agenda for change to grow. A collective agenda shapes group motivation and agreement to mobilise to act. At first, some might be motivated by the desire to change things for oneself in the early stages (private value) but shift to changing things for others (public value) might also drive this. Here theories of cooperation help to unpack the mechanisms and transitions at work. |
| Enactment  Creativity  Attainment | ‘Us’—‘We’  ► Shared decision-making  ► Contribution to something bigger than ‘I’ or ‘We’  ► Collective agency  ► Responsibility for change for others  ► Belonging to a ‘community’ | Empowerment and design theories:  As experience-based co-design progresses, new changes need to be enacted; without this nothing gets done. Enactment is dependent to some degree on creativity which enables people to bring their different experiences and skills to the co-design process. This might also mean bringing in others who can foster new ways of thinking creatively. Attainment is required for implementation. Here, empowerment and design theories provide a theoretical underpinning to examine these mechanisms and transitions. |
