# Supplementary material for: Evaluating the Co‐Design and Implementation of a Multicomponent Intervention to Improve Communication in Aged Care: A Nested Process Evaluation Protocol
Source: Health Expect. 2026 Jul 25;29(4):e70782. doi: 10.1111/hex.70782 (PMC13401143; doi:10.1111/hex.70782)
Supplement: Supplementary file 2 — Supporting File 2 [file HEX-29-e70782-s007.docx]

Your experiences of involvement in the *[removed for anonymization]* project

[Responses to questions 7-11 will appear on a five point Likert scale as follows. Icons will be coloured red, yellow and green to support understanding and “unsure” icon will be represented by two hands rather than a whole body]

| Disagree  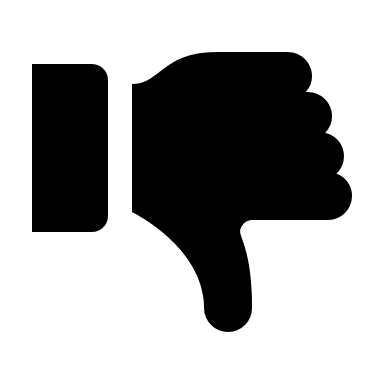 | Somewhat disagree  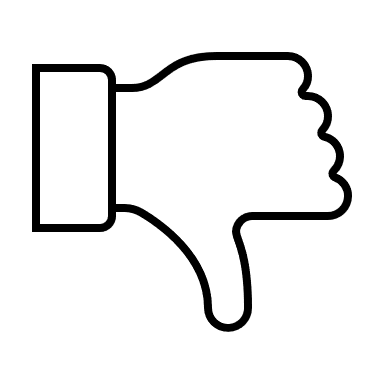 | Unsure  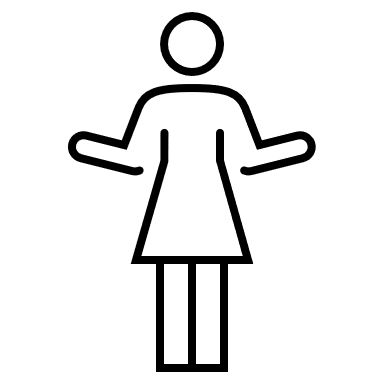 | Somewhat agree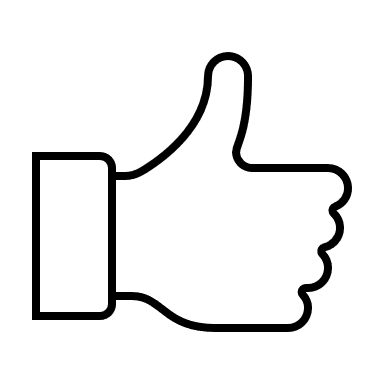 | Agree  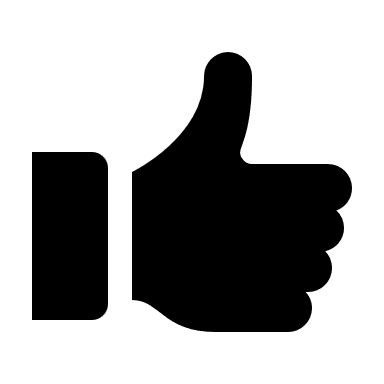 |
| --- | --- | --- | --- | --- |

1. Thinking about your participation in the [codesign workshop/user testing interview], please indicate the extent you agree or disagree with the following statements:
   1. I felt heard
   2. I gained a better understanding of others’ perspectives and experiences
   3. I was able to contribute to the best of my ability
   4. I felt a sense of shared control and ownership amongst those involved
   5. I felt we were working together to make a difference
2. Is there anything else you would like to share with us about your experience of participation in the [codesign workshop or user testing interview]? [free text response]
