# Supplementary material for: Evaluating the Co‐Design and Implementation of a Multicomponent Intervention to Improve Communication in Aged Care: A Nested Process Evaluation Protocol
Source: Health Expect. 2026 Jul 25;29(4):e70782. doi: 10.1111/hex.70782 (PMC13401143; doi:10.1111/hex.70782)
Supplement: Supplementary file 3 — Supporting File 3 [file HEX-29-e70782-s005.docx]

**Workstream:**

**Participant group:**

**Facilitator:**

**Date and time of codesign activity:**

**Date and time of observations:**

| Inputs | Mechanisms | Outcomes |
| --- | --- | --- |
| Experiential knowledge | Recognition | - Understanding - Respect - Feeling heard - Expressing needs |
| Sharing experiences | Dialogue | - Empathy - Change in beliefs - Agreement |
| Preparation for codesign | Cooperation | - Trust - Confidence - Self-Determination - Capacity to participate |
| Shared decision making | - Accountability - Mobilisation | - Shared commitment - Change in attitudes - Control & Ownership |
| Codesign of solutions | - Enactment - Creativity - Attainment | - Advocacy - Participatory Collaboration - Empowerment |
| General reflections: | | |

Adapted from: Palmer, V. J., Weavell, W., Callander, R., Piper, D., Richard, L., Maher, L., Boyd, H., Herrman, H., Furler, J., Gunn, J., Iedema, R., & Robert, G. (2019). The Participatory Zeitgeist: an explanatory theoretical model of change in an era of coproduction and codesign in healthcare improvement. *Medical Humanities*, *45*(3), 247. <https://doi.org/10.1136/medhum-2017-011398>
