# Supplementary material for: Evaluating the Co‐Design and Implementation of a Multicomponent Intervention to Improve Communication in Aged Care: A Nested Process Evaluation Protocol
Source: Health Expect. 2026 Jul 25;29(4):e70782. doi: 10.1111/hex.70782 (PMC13401143; doi:10.1111/hex.70782)
Supplement: Supplementary file 4 — Supporting File 4 [file HEX-29-e70782-s002.docx]

*N.B. These questions are intended to provide a rough guide of topics to be covered and do not preclude the interviewer from pursuing lines of enquiry that emerge from participant responses that may contribute to the overall research question and aims.*

For the purpose of today’s interview, I’d like to focus our conversation on your experiences of participating in the *[removed for anonymization]* project. The researchers conducting the project are keen to know what worked and didn’t work for people who were involved. They will not know what you said, specifically, so please feel free to speak freely.

1. To get us started, can you tell me what motivated you to get involved with the project?

*Prompt, if needed:*

- 1. How would you describe your overall experiences of communication in your aged care service [prompt with demographic info provided]?
  2. What difference did you hope to make?

1. How have you been involved in the *[removed for anonymization]* project so far?

*Prompt, if needed:*

- - 1. How did you find out about the project?
    2. How have the project team sought your input?

1. What has your experience of involvement in the project been like so far?

*Prompt, if needed:*

- - 1. Can you describe an example of when you felt you were meaningfully involved in the process?
    2. What do you think the facilitators (or other participants) did that supported your contribution?
    3. Were there any instances where you felt you weren’t meaningfully involved in the process?
    4. What do you think made the difference that time?

1. Has your experience with the *[removed for anonymization]* project changed anything for you, on a personal level?

*Prompt, if needed:*

1. Has your perspective changed? How?
2. Do you feel differently than you did before? How?
3. Are you doing anything new or differently since participating?
4. The project is aiming to develop:
   - A profiling tool for identifying communications needs of aged care recipients
   - Communication partner training for aged care workers
   - Guidelines for improving communication in aged care services
5. Do you think these changes will improve communication in your aged care service? *Prompt, if needed:* Why is that?
   - 1. Is there anything beyond these changes that you see as important in improving communication in aged care services? *If so*: Did you get to say this in the [codesign workshop/user testing interview]?
6. If there was one thing you think might improve future codesign activities in similar projects, what would it be? *Prompt for reasoning.*
7. Is there anything important you’d like to come back to, or anything else you would like to share?
