# Supplementary material for: Evaluating the Co‐Design and Implementation of a Multicomponent Intervention to Improve Communication in Aged Care: A Nested Process Evaluation Protocol
Source: Health Expect. 2026 Jul 25;29(4):e70782. doi: 10.1111/hex.70782 (PMC13401143; doi:10.1111/hex.70782)
Supplement: Supplementary file 5 — Supporting File 5 [file HEX-29-e70782-s008.docx]

*N.B. These questions are intended to provide a rough guide of topics to be covered and do not preclude the interviewer from pursuing lines of enquiry that emerge from participant responses that may contribute to the overall research question and aims.*

For the purpose of today’s focus group, I’d like to focus our conversation on changes at your aged care service related to the *[removed for anonymization]* project. The project sought the input of aged care recipients, families and staff to develop:

- A tool for identifying communications needs of aged care recipients
- Training for aged care workers
- Guidelines for improving communication in aged care services

1. To get us started, I’d like to get a sense of how involved you have been in the development of these resources. Were you involved in any co-design interviews, workshops or testing of the resources?
   1. *If so:* Can you tell me a bit about that?
   2. *If not:* Were you aware the project was happening before being invited to participated in the trial?
2. What has your experience of communication with staff been like in your aged care service in the past?

*Prompt if needed:*

1. What makes communication with staff easy in your aged care service?
2. What impact does good communication have on you?
3. What makes communication with staff difficult in your aged care service?
4. What impact does poor communication have on you?
5. Have you noticed any changes in recent months in communication with staff in your aged care service? *Prompt if needed:* Has anything changed in your day-to-day care?

*If yes:*

- - 1. How have things changed for you?
    2. What has that change meant for you?
    3. How have you felt about the change?

1. If there was one thing you think might support better communication between staff and people receiving care in your aged care service, what would it be? *Prompt for reasoning.*
2. Is there anything important we haven’t covered anyone would like to share?
