# Supplementary material for: Evaluating the Co‐Design and Implementation of a Multicomponent Intervention to Improve Communication in Aged Care: A Nested Process Evaluation Protocol
Source: Health Expect. 2026 Jul 25;29(4):e70782. doi: 10.1111/hex.70782 (PMC13401143; doi:10.1111/hex.70782)
Supplement: Supplementary file 6 — Supporting File 6 [file HEX-29-e70782-s010.docx]

*N.B. These questions are intended to provide a rough guide of topics to be covered and do not preclude the interviewer from pursuing lines of enquiry that emerge from participant responses that may contribute to the overall research question and aims.*

For the purpose of today’s focus group, I’d like to focus on the implementation of changes at your aged care service related to the *[removed for anonymization]* project. The project sought the input of aged care recipients, families and staff to develop:

- A tool for identifying aged care recipients who need extra support with communication
- Training for aged care workers in providing support with communication
- Guidelines for improving communication in aged care services

1. To get us started, I’d like to get a sense of how involved you have been in the development of these resources. Were you involved in any co-design interviews, workshops or testing of the resources?
   1. *If so:* Can you tell me a bit about that?
   2. *If not:* Were you aware the project was happening before being invited to participated in the trial?
2. Have you had the opportunity to use any of the resources developed by the *[removed for anonymization]* project?
   1. *If so:* Which resource did you use? What supported you to do that? How did you find it?
   2. *If not:* What has prevented you from being able to use the resources?
3. Has anything changed for you at work since using the *[removed for anonymization]* resource/s?
   - 1. *If yes:* How have things changed? Who else has been impacted by this change? How have you felt about the change?
     2. *If no:* Why do you think that is?
4. If there was one thing you think might improve the use of the *[removed for anonymization]* resources, what would it be? *Prompt for reasoning.*
5. Beyond the resources developed by the *[removed for anonymization]* project, is there anything else you think could be effective in improving communication in aged care services?
6. Is there anything important we haven’t covered anyone would like to share?
