# Supplementary material for: Evaluating the Co‐Design and Implementation of a Multicomponent Intervention to Improve Communication in Aged Care: A Nested Process Evaluation Protocol
Source: Health Expect. 2026 Jul 25;29(4):e70782. doi: 10.1111/hex.70782 (PMC13401143; doi:10.1111/hex.70782)
Supplement: Supplementary file 8 — Supporting File 8 [file HEX-29-e70782-s001.docx]

The following questions are to enable description of the overall participant group in the *[removed for anonymization]* evaluation and exploration of the similarities and differences between groups. In presentations or publications of the findings, this information will be reported in such a way to protect the anonymity of your feedback.

1. Which of the following statements describe you?

I am a recipient of aged care services.

I am a family member or informal carer of one or more aged care recipients.

I am employed by an aged care service to deliver direct care to aged persons. My current job title is:__________________________________________________________________

I manage staff providing aged care services.

I am a healthcare professional with expertise in communication difficulties (e.g., speech pathologist/therapist, audiologist)

If so, what is your specific job title:____________________________________________________

I am a member of an advocacy group or organisation

Other. Please specify:________________________________________________________________

- 1. *If employed by an aged care service or a health care professional:*

How long have you been employed in this role?

Less than one year

1-5 years

6-10 years

11-15 years

16 years or more

1. Which of the following describes the aged care service you have had your most recent experiences with? Select as many as apply.

A residential aged care service (e.g., short term or respite care including day respite, nursing home).

An in-home aged care service (e.g., care in private homes)

- 1. *If an aged care recipient:* In what post code do you receive aged care services? ___________
  2. *If a supporter:* In what postcode does the person you support receive aged care services? ___________
  3. *If employed by an aged care service or a health care professional:* In what postcode/s do you mainly work? ___________

1. What is the highest level of education you have completed?

Year 7 or equivalent (i.e., completed primary education)

Year 10 or equivalent (i.e., completed junior certificate)

Year 12 or equivalent (i.e., completed secondary education)

Vocational certificate or diploma (e.g., TAFE)

Undergraduate/Bachelor degree

Post-graduate degree

- 1. *If employed by aged care service or health care professional:*

What is your highest level of education relevant to your current role?

I have not completed any formal education relevant to my role.

Vocational certificate or diploma.

Please specify:____________________________________________________________________

Undergraduate/Bachelor degree

Please specify:____________________________________________________________________

Post graduate degree

Please specify:____________________________________________________________________

1. Do you speak a language other than English at home?

Yes

No

- 1. *If “yes”:*

Which of the following statements best describe your confidence in using English to communicate in your aged care service?

I always feel confident communicating in English with others (e.g., staff or aged care recipients) in my aged care service.

I sometimes feel confident communicating in English with others (e.g., staff or aged care recipients) in my aged care service.

I do not feel confident communicating in English with others (e.g., staff or aged care recipients) in my aged care service.

1. Are you Aboriginal or Torres Strait Islander?

Yes, I am Aboriginal

Yes, I am Torres Strait Islander

Yes, I am both Aboriginal and Torres Strait Islander

No, I am neither Aboriginal nor Torres Strait Islander
