# Supplementary material for: Evaluating the Co‐Design and Implementation of a Multicomponent Intervention to Improve Communication in Aged Care: A Nested Process Evaluation Protocol
Source: Health Expect. 2026 Jul 25;29(4):e70782. doi: 10.1111/hex.70782 (PMC13401143; doi:10.1111/hex.70782)
Supplement: Supplementary file 9 — Supporting File 9 [file HEX-29-e70782-s003.docx]

**Workstream:**

**Activity (e.g., workshop, user testing):**

**Participant group:**

**Facilitator:**

**Date and time of codesign activity**

**Date and time of fieldnote:**

| Prompts | Facilitator comments |
| --- | --- |
| Contextual information:   - Location (e.g., online/in person/hybrid) - Notable environment features - How many people in the room/online - Others present (e.g., supporters) |  |
| Participant engagement:   - Who was engaged/disengaged? - Atmosphere - Non-verbal behaviours |  |
| Review of input provided by co-designers   - Input that was new or unexpected - Topics on which there was agreement/disagreement |  |
| Reflection on your facilitation:   - What worked well/not well - Thoughts for next time |  |

Adapted from: Phillippi J, Lauderdale J. A Guide to Field Notes for Qualitative Research: Context and Conversation. Qualitative Health Research. 2018;28(3):381-388. doi:10.1177/1049732317697102
