# Supplementary material for: Evaluating the Co‐Design and Implementation of a Multicomponent Intervention to Improve Communication in Aged Care: A Nested Process Evaluation Protocol
Source: Health Expect. 2026 Jul 25;29(4):e70782. doi: 10.1111/hex.70782 (PMC13401143; doi:10.1111/hex.70782)
Supplement: Supplementary file 10 — Supporting File 10 [file HEX-29-e70782-s011.docx]

**Participant/Focus group identifier:**

**Interviewer/facilitator:**

**Date and time of observations:**

**Date and time of fieldnote:**

| Contextual information:   - Location - Season/weather (typical or not) - Holidays or major events - Environment interview/focus group held - Others present in the room   - their relationship to participant/s or research   - reason for participation |  |
| --- | --- |
| Interactions:   - Participant/s appearance - Participant/s demeanour - Relevant demographic information shared by participants |  |
| Interview/focus group: Overview   - Atmosphere - Non-verbal behaviours - Overall depth of content |  |
| Interview/focus group: Review of each question/topic area   - Interactions amongst participants - Depth of response - Value of question - Tentative thoughts on codes and category |  |
| Personal reflection:   - Overall thoughts - Setting - Potential biases - Reflection on your interviewing/facilitation - Thoughts on questions - Changes for future interviews/focus groups - Tentative codes - Saturation |  |

Adapted from: Phillippi J, Lauderdale J. A Guide to Field Notes for Qualitative Research: Context and Conversation. Qualitative Health Research. 2018;28(3):381-388. doi:10.1177/1049732317697102
