# Supplementary material for: Evaluating the Co‐Design and Implementation of a Multicomponent Intervention to Improve Communication in Aged Care: A Nested Process Evaluation Protocol
Source: Health Expect. 2026 Jul 25;29(4):e70782. doi: 10.1111/hex.70782 (PMC13401143; doi:10.1111/hex.70782)
Supplement: Supplementary file 11 — Supporting File 11 [file HEX-29-e70782-s004.docx]

**Aged care service identifier:**

**Site identifier:**

**Researcher recording field note:**

**Date and time of site visit:**

**Date and time of fieldnote:**

| Contextual information:   - Location - Season/weather (typical or not) - Holidays or major events - Areas of site visited - Others present during visit |  |
| --- | --- |
| Interactions:   - Staff/aged care recipients’/carers appearance - Staff/aged care recipients’/carers demeanour - Relevant demographic information shared by staff/aged care recipients’/carers (e.g., role, qualifications, CALD, ATSI) |  |
| Overview of visit:   - Atmosphere - Non-verbal behaviours - Overall depth of discussions |  |
| Observations relevant to implementation of [removed for anonymization] intervention components (RE-AIM framework domains) | Reach: |
|  | Effectiveness: |
|  | Adoption: |
|  | Implementation: |
|  | Maintenance: |
| Personal reflection:   - Overall thoughts - Reflection on my facilitation of site visit - Comparison with data collected - Questions for future site visits or data collection |  |

Adapted from: Phillippi J, Lauderdale J. A Guide to Field Notes for Qualitative Research: Context and Conversation. Qualitative Health Research. 2018;28(3):381-388. doi:10.1177/1049732317697102
